# MMP-9 as a diagnostic salivary biomarker for early detection of oral cancers: systematic review and meta-analysis

**DOI:** 10.1186/s12903-025-07513-x

**Published:** 2026-01-03

**Authors:** Aqsa Shaukat, Saleha Nisar, Mehmood Asghar, Muhammad Kaleem, Muhammad Sohail Zafar

**Affiliations:** 1 Department of Science of Dental Materials, AM college, NUMS, Rawalpindi, Pakistan; 2 Department of Clinical Sciences, College of Dentistry, Ajman University, Ajman, United Arab Emirates; 3 Centre of Medical and Bio-allied Health Sciences Research, Ajman University, Ajman, United Arab Emirates; 4 School of Dentistry, University of Jordan, Amman, Jordan

**Keywords:** Salivary biomarkers, Oral squamous cell carcinoma, OSCC, Diagnostic accuracy studies, Matrix-metalloproteinase-9, MMP-9

## Abstract

**Objectives:**

The intention of this investigation was to evaluate the diagnostic performance of salivary MMP-9 enzyme for rapid point of care testing of early stage (I, II) oral cancer. Diagnosis at early pre-malignant stages will help in making clinical decisions for timely intervention.

**Materials and methods:**

Original diagnostic test accuracy (DTA) studies of any design, on patients with lip and oral cavity cancers investigating salivary MMP-9 levels as an index test and histopathological confirmation via biopsy as a reference test were eligible for inclusion. Similar studies on serum or blood were excluded. Besides, the case reports/case series, reviews, letters, book chapters, conference abstracts, in vitro, and animal studies were all excluded. Non-English papers were not excluded. The databases searched from Jan 2014 to Aug 2024 were PubMed, SCOPUS, Cochrane Library, Science Direct, and Google Scholar, without language restrictions. QUADAS-2 was used to assess the risk of bias and concerns regarding applicability. Meta-analyses were performed using a bivariate random-effects model.

**Results:**

Out of 18 articles identified, 7 studies with 795 patients were included. The pooled sensitivity, specificity, mean salivary MMP-9 levels, and the Summary Receivers Operating Curve were done using REVMAN 5.4 and a web application (Meta-DiSc 2.0). Overall, the pooled sensitivity, specificity, and Area under the curve were 0.98, 0.96, and 0.97, respectively with 95% confidence interval between 0.85 and 1. However, with high heterogeneity and risk of bias together with low applicability concern we need additional pragmatic designed research to back up its use in screening and diagnostic applications.

**Conclusion:**

Within the limitations of the study, salivary MMP-9 has merit as a biomarker for identification of early-stage (I, II) oral cancer.

**Supplementary Information:**

The online version contains supplementary material available at 10.1186/s12903-025-07513-x.

## Introduction

According to GLOBOCAN 2022, Lip and oral cavity cancer (LOC) is the 16th most diagnosed neoplasm (C00-C06) overall. Almost 389,485 new cases of oral cancer were diagnosed, and over 188,230 deaths were reported worldwide [1]. Cancers of the lip and oral cavity encompass malignancies affecting the lip, tongue, and oral cavity, primarily developing from epithelial cells, with approximately 90% classified as oral squamous cell carcinoma (OSCC) [2]. The cumulative risk for males and females worldwide is 21.79% and 18.5% [1].

The occurrence of OSCC is correlated with various risk factors, including tobacco use (chewing or smoking), alcohol consumption, intake of foods high in nitrosamines, infection with human papillomavirus, environmental exposures such as ultraviolet radiation, and dietary deficiencies resulting from insufficient consumption of fruits and non-starchy vegetables [3]. The incidence of oral cancers can be reduced by minimizing exposure to risk factors or by timely screening for potentially malignant oral disorders (OPMDs), including leukoplakia, erythroleukoplakia, oral lichen planus, and oral submucous fibrosis [4]. Therefore, early diagnosis is crucial for improving the prognosis and patient outcomes. Unfortunately, in most cases, by the time it is diagnosed, malignancy has already reached its advanced stages. Traditional method of diagnosing OSCC is to perform a surgical resection of the suspected tissue and doing a histopathological study on that tissue. It is a gold standard diagnostic procedure for identifying oral cancer [5]. This modality has its limitations as well, such as patient discomfort, invasiveness, and potential sampling errors related to subjective interpretation. Besides, often times the histopathological picture is inconsistent with clinical findings [5, 6]. For these reasons, there is a need to explore alternate diagnostic methods [7, 8].

A biological marker is an indicator of a state, condition, or process in a biological system. Unlike any medical symptom, which are subjective experiences patients report, biomarkers are measurable or objective medical signs [9], hence considered chemical fingerprints. They can be proteins, nucleic acids, metabolites, or other molecules found in body fluids. Using biomarkers as a diagnostic tool can provide objective results while enhancing patient comfort and compliance. The neoplastic changes and their effects are seen quite early biochemically compared to clinical diagnosis or histopathology [7]. Hence, by using biomarkers we can identify oral cancer at earlier stages (I, II) before progression to advanced stages.

The biomarkers that can be used for OSCC diagnosis are released in biofluids like blood, urine, sweat, and saliva [7, 10]. Saliva is described as a mirror of the body, providing a comprehensive snapshot of overall health [11]. Its use as a diagnostic medium for the biomarker offers a promising alternative to tissue biopsies. Its collection is easy, non-invasive, fast, and cost-effective [12]. Based on the disease state, salivary biomarkers can be divided into prediction, detection, diagnostic, and prognostic biomarkers [13]. Researchers have explored salivary factors as biomarkers for OSCC and potentially malignant disorders (OPMD). Analysis of salivary proteomics, genomics together with the variation in their levels, may provide us insights into progression of oral cancer, its prognosis and post-treatment follow-ups [12, 14].

A gelatinase enzyme matrix metalloproteinase-9 (MMP-9) breaks down denatured collagen or gelatin under physiological process involving tissue remodeling like wound repair, formation and development of blood vessels and embryo. It particularly degrades type-IV collagen, which is an important basement membrane component [15, 16]. The MMP-9 regulation is disturbed in other pathological conditions, such as diabetes mellitus, neurodegenerative diseases, renal pathologies, and cardiac conditions [17], however in cancers MMP-9 enzyme regulation is lost at multiple levels such as during its transcription, activation, interaction with extracellular matrix (ECM), inhibition by tissue inhibitors for matrix metalloproteinase (TIMPs) or during its elimination from the ECM. Invading tumor cells secrete dysregulated proteases like MMPs, which degrade ECM and basement membrane. This increase in proteases facilitates cell motility, changes cell-cell adhesion properties, rearranges ECM, suppresses programmed cell death, and reorganizes their cytoskeleton. MMPs like MMP-2 and 13 are also raised in OSCC and increase with disease progression [17]. MMP-9 has been identified as a helpful early diagnostic biomarker that detects tumor metastasis and prognosis in OSCC patients [18]. Furthermore, a study linked MMP-9 expression with tumor invasion and lymph node involvement in OSCC. Interestingly, there are also reports that the level of MMP-9 doesn’t seem to be linked to the stage of the tumor or the lymph nodes., but rather as a prognostic factor for poor survival in head and neck carcinoma [14]. This lack of consensus highlights the uncertainty surrounding MMP-9’s role as a prognostic or diagnostic biomarker.

Existing literature has not exclusively concentrated on its role as a biomarker for the diagnosis of oral cancer in its early stages (I, II). Utilizing systematic review protocols and methodologies, we have collected original research articles about MMP-9 in the context of oral cancer, thereby offering a thorough synthesis of the existing knowledge on this topic. Our objective was to present an overview of the potential significance of salivary MMP-9. Therefore, the primary aim of this review was to investigate the relationship between salivary MMP-9 biomarkers and oral cancer and its diagnostic accuracy for the oral cancers in its early stages.

## Materials and methods

The review was conducted thoroughly following the PRISMA Checklist [19] to ensure a systematic approach. The protocol was registered at PROSPERO as CRD 42,024,573,382.

### Search protocols

The search protocols were defined, and a PICO format review question was formulated. The focused question was, “Can salivary MMP-9 levels be used for diagnosis of early-stage (I, II) oral cancer in adult patients?”. The study will evaluate salivary MMP-9 diagnostic performance (outcome) in adult patients with oral cancer (population) as compared to healthy patients (control) using ELISA (intervention).

### Eligibility criteria

The diagnostic test accuracy (DTA) studies of any design, measuring salivary MMP-9 levels in adult patients, were included in this review. However, studies testing MMP levels other than MMP-9 or those testing in body fluids other than saliva were excluded. The case reports/case series, reviews, letters, book chapters, conference abstracts, in vitro, and animal studies were also excluded. Non-English papers were not excluded.

### Information sources and search strategy

Detailed individual search strategies were developed for the Cochrane Library, MEDLINE, Science Direct, and SCOPUS databases. The search covered all articles published between January 2014 and August 2024, without language or other filters. References were manually maintained, duplicates eliminated, and further references reviewed in selected papers.

### The search string

A search string was defined according to PICO criteria [19] Various outcomes including salivary MMP-9 levels, Area Under the Curve (AUC), sensitivity (SEN), and specificity (SPE) were measured. The target population was adults aged 20–65 with oral cancer, oral potentially premalignant disorders (OPMD) or suspicious lesions undergoing diagnostic methods like in situ zymography or ELISA. The histopathological confirmation with biopsy is a gold standard reference test for oral cancer against a healthy control group. The string was fine-tuned for every database (Table 1).

**Table 1:** Electronic databases and respective keyword combinations used

Database	Search string	Date
Science Direct	(((((“oral cancer”) OR “oral squamous cell carcinoma” OR (“OSCC”)) AND (“saliva biomarkers”)) AND “ELISA” OR “in situ zymography” AND (“matrix metalloproteinases”)) OR (“MMP”) AND “diagnostic accuracy”	12-7-24
MEDLINE	(((((“oral cancer”) OR “oral squamous cell carcinoma” OR (“OSCC”)) AND (“saliva biomarkers”)) AND “ELISA” OR “in situ zymography” AND (“matrix metalloproteinases”)) OR (“MMP”) AND “diagnostic accuracy”	12-7-24
Google Scholar	(((((“oral cancer”) OR “oral squamous cell carcinoma” OR (“OSCC”)) AND (“saliva biomarkers”)) AND “ELISA” OR “in situ zymography” AND (“matrix metalloproteinases”)) OR (“MMP”) AND “diagnostic accuracy”	12-7-24
Cochrane library	“Oral cancer” OR “oral squamous cell carcinoma” OR “OSCC” AND “saliva biomarkers” AND “ELISA” OR “in situ zymography” AND “matrix metalloproteinases” OR “MMP” AND “diagnostic accuracy”	12-7-24
SCOPUS	Oral AND cancer OR OSCC AND salivary AND biomarkers OR tumor, biomarkers AND matrix metalloproteinases OR MMP AND diagnostic accuracy	13-7-24
Web of Science	Oral AND cancer OR OSCC AND salivary AND biomarkers OR tumor, biomarkers AND matrix metalloproteinases OR MMP	13-7-24

### Screening of data

The extraction of data was carried out by three researchers. In the first step, AS selected the studies independently. The titles and abstracts of the references were assessed. The selected papers met the inclusion criteria based on their titles and abstracts. Studies that did not match the inclusion criteria were excluded. SN and MA evaluated the screening results.

In the second stage, entire papers were reviewed to determine which ones reported sensitivity and specificity or offered data that permitted these diagnostic evaluations to be made. AS also carefully examined the reference lists of all included papers. Any differences during either phase were resolved through discussion and consensus among the three reviewers (AS, SN and MA).

### Extraction of data

#### Data processing

The extraction process involved three researchers. AS gathered the necessary information from the chosen articles. SN verified the collected data and ensured its accuracy. AS obtained the essential information from the chosen articles. SN verified the collected data to guarantee its accuracy. Any discrepancies during any stage was resolved through discussion among AS, SN, and MA.

#### Data items

The information from included studies like authors name, country, year of publication, sample size, reference/index test and outcomes reported were documented. Outcome measures include mean MMP-9 levels (ng/ml), sensitivity (SEN), specificity (SPE), and area under the curve (AUC). If any required data were missing, no efforts were made to contact the authors for the missing information.

### Qualitative and quantitative assessment

#### Analysis of bias

The Quality Assessment Tool for Diagnostic Accuracy Studies (QUADAS) was used to assess the bias. Reviewers (AS) and (SN) answered each question in the tool as either “yes,” “no,” or “unclear” and evaluated the quality of each study included.

#### Meta-analysis

A meta-analysis was conducted to evaluate the ability of the MMP-9 to distinguish OSCC patients from controls. The web application Meta DiSc 2.0 [20], was utilized to create a summary receiver operating characteristic (SROC) graph. At the same time, REVMAN version 5.4 was employed for generating forest plots as part of the meta-analysis.

## Results

**Fig. 1:**
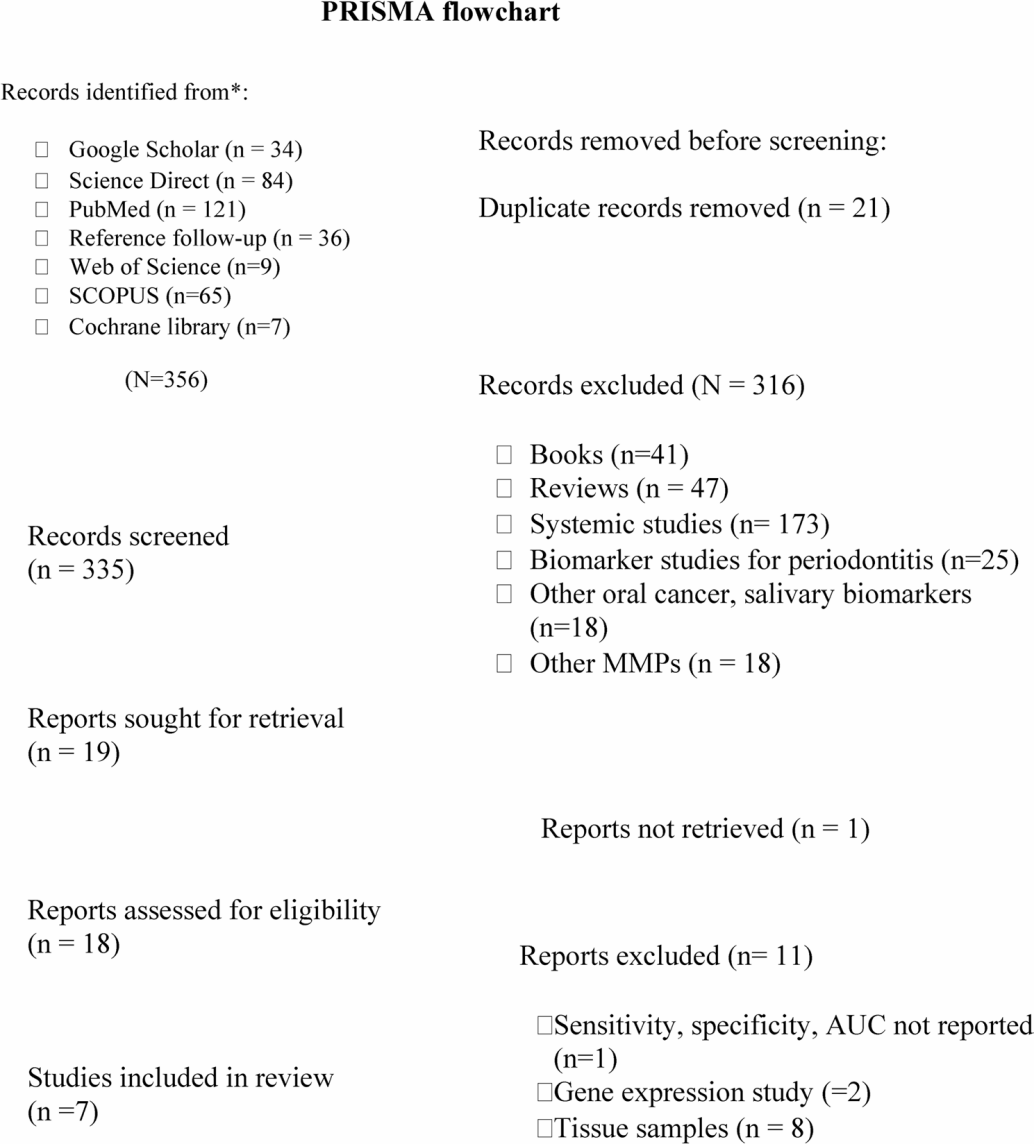
PRISMA Flowchart

A total of 356 papers were identified from the databases. After removing the duplicates (*n* = 21), the electronic search yielded 335 studies. Out of these, 316 were excluded after title and abstract screening. A total of 18 reports were sought for retrieval and then assessed for eligibility. Only 7 studies [21–27] met the specified inclusion criteria in this review, encompassing 795 patients (398 controls, 209 OSCC, 166 OPMD, and 22 smokers). A flow chart detailing the process of identifying, including, and excluding studies is shown in Fig. 1.

### Study characteristics

The characteristics of the studies included in this review are shown in Table 2. The included papers were from Afghanistan, Egypt, Germany, India, Korea, and Russia between 2017 and 2023. All these studies were two-gate exploratory DTA studies [28, 29]. Patients were grouped into case and control groups. The case group included OSCC and OPMD patients. The OSCC patients were from all stages (I -IV). Only one study reported MMP-9 according to OSCC grading [26]. The OPMD reported were oral submucous fibrosis (OSF) leucoplakia, erythroplakia and papilloma. The tongue and buccal mucosa were more frequently reported sites [21, 26]). The details on TNM stages or anatomical subsites are mentioned in supplementary table S1. All studies used the ELISA immune assay method to measure salivary MMP-9 levels, while no study used in situ zymography. The cut-off/threshold values were not pre-specified for any of the studies, and few studies reported any cut-off values [25–27]. All studies reported AUC, SEN, SPE, and mean MMP-9 values except for two with missing data, either mean values [23] or SEN/SPE [25]. The gold standard or reference test for all studies was histopathological confirmation through biopsy; however, one study also used CT-PET. Salivary MMP-9 levels were considered an index test and reported in ng/ml, except for one study where no scale was mentioned [23].

**Table 2:** Study characteristics

Author names/ countries	Type and method of saliva collection	Sample size	Cases/ control	Index test (MMP-9ng/ml)	Cut off value (ng/ml)	SEN	SPE	AUC
Ghallab and Shaker 2017 [21] (Egypt)	USWS by Passive drooling method	45	15 OSCC	1642.5 ± 1498.9	260	100%	100%	1
			15 OPMD	275.81 ± 30.88	214	100%		0.99
			15 control	108.44 ± 31.43	318.7			
Kochurova et al. 2017 [27] (Russia)	Not specified	122	57 PL	403 ± 69.8	NR	83.30%	53.30%	0.5
			65 control	388 ± 125.80				
Pazhani et al. 2023 [26] (India)	USWS by Passive drooling method	102	34 OSCC	50.9 ± 5.7	NR	100%	26.70%	NR
			34 PL	31.6 ± 6.0				
			34 controls	16.2 ± 4.8				
Peisker et al. 2017 [23] (Germany)	Stimulated saliva samples	60	30 OSCC	NR	0.014	100%	26.70%	0.698
			30 controls					
Shin et al. 2021 [24] (Seoul/Korea)	USWS by Passive drooling method	318	106 OSCC	511.86 ± 0.12	120.9			
			212 controls	29.27 ± 0.08		89.60%	100%	0.96
Smriti et al. 2020 [22] (India/ Afghanistan)		88	24 OSCC	387.87 ± 126.51	214	100%	59%	0.917
			20 OPMD	339.86 ± 115.7	205.8	100%	54%	0.852
	USWS		22 smokers					
			22 controls	213.91 ± 67.11				
Thiruvalluvan et al. 2021 [25] (India)	Unstimulated salivary samples	60	20 OSMF	9.42 ± 0.859	NR	NR	NR	0.9
			20 OL	10.59 ± 0.859				
			20 controls	2.96 ± 0.859				

All studies used ELISA and “histopathology following biopsy” as a reference test.

### Risk of bias

**Fig. 2:**
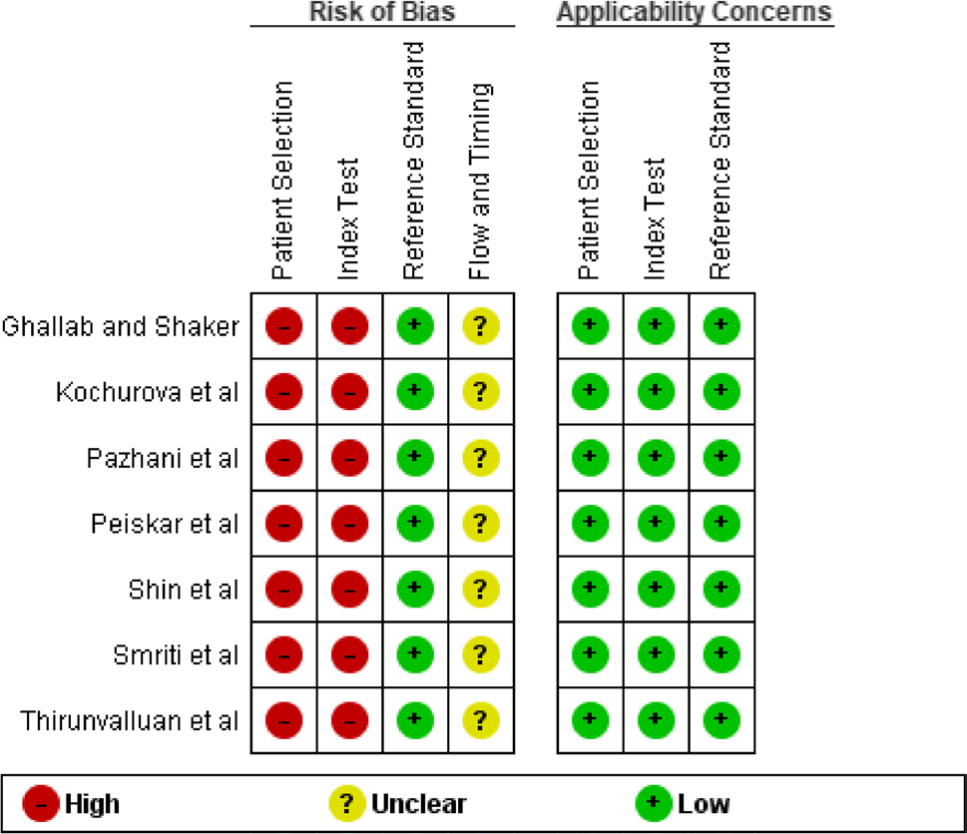
Quality assessment using the QUADAS-2 tool for individual diagnostic accuracy studies

**Fig. 3:**
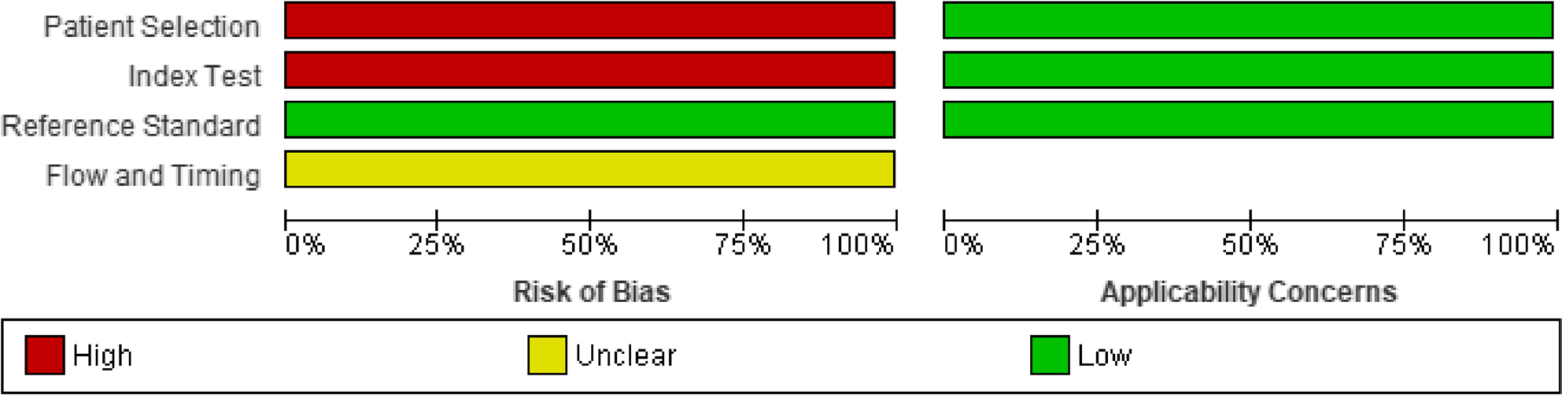
Graph for risk of bias and applicability concern, QUADAS-2 tool across diagnostic accuracy studies

The QUADAS-2 tool [30] was used for qualitative assessment. Figures 2 and 3 mention the risk of bias and applicability concerns for individuals and across studies.

The study designs, as reported, were prospective [23, 27], cross-sectional [22], observational [26], case-control [24], and clinical trial [21, 25]. All studies used two gates for patient selection, i.e., case and control groups were selected on different criteria, instead of using a single pool of patients with the same selection criteria. It was also based on prior knowledge of reported OSCC, which was later confirmed through histopathology, and inappropriate exclusions weren’t mentioned either. No study mentioned whether both index and reference test results were interpreted with/without knowledge of the results of the other one. The reference standard used was likely to classify the target condition, so overall bias in this section was reported to be low. The threshold/cut-off values were reported by [21–24] but were not pre-specified. These studies did not report the fourth domain, which discusses the flow and timing of both tests. The concerns for applicability were low, as the study population used in individual studies was the same as the review question. Overall, there was a high risk of bias, which should be interpreted by keeping exploratory designs in sight. Cohen’s kappa score was calculated using an online tool [31]. It was 0.35, which showed fair agreement.

### Results of individual studies

All included studies emphasize the significance of salivary MMP-9 as a biomarker for early-stage oral cancer. Ghallab and Shaker [21] noted its use in screening and diagnosing early cancer, though it’s not a replacement for established diagnostic tools. Shin et al. [24] found that salivary MMP-9 levels may improve outcomes in OSCC patients, including mortality and morbidity. Smriti et al. [22] consider it an early detection diagnostic tool, but not for recurrence follow-up. A non-intrusive saliva-based screening kit could aid in identifying OSCC and OPMD patients. Pazhani et al. [26] observed higher salivary MMP-9 levels in poorly differentiated OSCC, suggesting it as an alternate marker for cancerous transformation. Salivary diagnostics for OSCC are promising due to the direct contact between oral cancer lesions and saliva, making it non-invasive and suitable for population screening, especially in remote areas [23, 27]. Thiruvalluvan et al. [25] found significant MMP-9 expression in oral leukoplakia and OSMF as compared to control, considering it a reliable biomarker for assessing OPMD malignant transformation. However, they reported an insignificant difference in expression between the two types.

### Meta-analysis

**Fig. 4:**
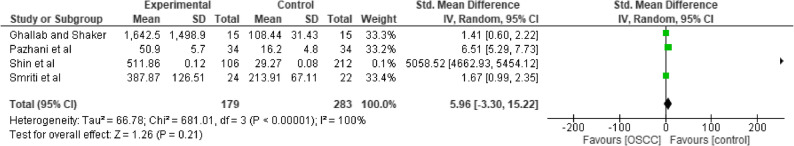
Forest plot of salivary MMP-9 in OSCC

**Fig. 5:**
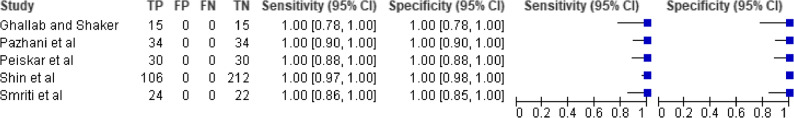
Sensitivity and specificity plot for salivary MMP-9 in OSCC

**Fig. 6:**
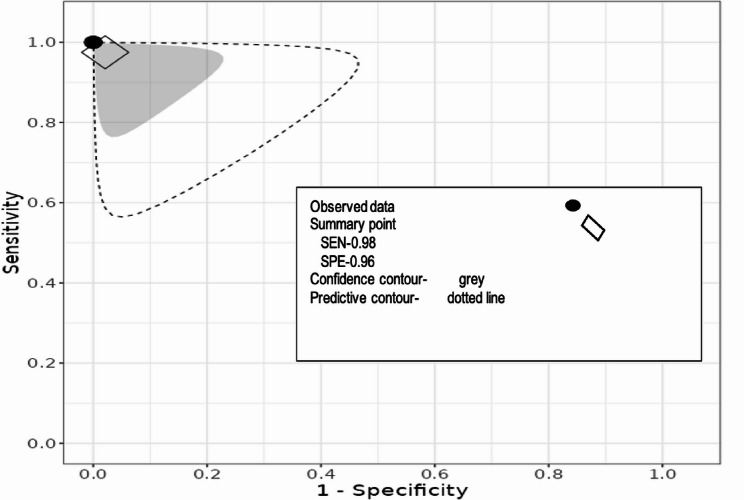
Summary Receiver Operators Curve (SROC) for salivary MMP-9 in OSCC

The forest plots for MMP-9 values for OSCC are shown in Fig. 4. The overall effect size was SMD 5.96 with 95% confidence interval − 3.30 to 15.22. The heterogeneity or I^2^ was 100% while p-value was 0.21. The quantitative synthesis for sensitivity and specificity is shown in Fig. 5. The pooled sensitivity and specificity were consistently higher, 1.00 and 1.00, respectively with 95% confidence interval between 0.85 and 1. The summary ROC graph (Fig. 6) shows the summary operating point (black point), confidence contour (grey), and prediction contour (dotted line). Since, the number of included studies were less than 10 the publication bias testing was not pursued.

## Discussion

The accuracy of diagnostic tests plays a pivotal role in clinical decision-making, influencing patient management and healthcare outcomes. The rapid diagnostic tests, due to their speed, convenience, and portability, are preferred over routine laboratory processing. In this systematic review, we evaluated the diagnostic performance of salivary MMP-9 enzyme for rapid testing of oral cancer in early stages (I, II). We synthesized evidence from multiple studies to provide a comprehensive assessment of its sensitivity and specificity. Our findings highlight key methodological considerations, clinical implications, and potential limitations inherent in the evaluation of diagnostic test accuracy.

Salivary MMP-9 is an enzyme protein involved in multiple cancer-related processes. It cleaves surface proteins, degrades ECM, and alters cell-to-cell and cell-to-ECM interactions [32]. Its involvement in other systemic conditions has also been reported. Its serum and, hence, salivary levels may increase in not only inflammatory conditions like diabetes, autoimmune diseases, and arthritis but also in hypertension, atherosclerosis, and myocardial infarction [33]. MMP-9 involvement in cancers has been reported extensively, such as in osteosarcoma, giant cell bone tumor, and other cancers, such as hepatocellular cancer, lung cancer, cervical cancer, ovarian cancer, and breast cancer [32], and specifically oral cancer [18, 34, 35]. Approximately 30% of the proteins in plasma are also present in saliva [36, 37], which suggests significant links between the salivary proteome and various bodily systems. For example, tumor necrotic factor-alpha is released in the serum and saliva of OPMD patients [32]. With more advanced analytical techniques, saliva can be a valuable resource for translating biomarker studies to point-of-care diagnostic devices, particularly in dental and personalized medicine [36, 37].

The present systematic review evaluated the potential role of MMP-9 in determining its diagnostic significance as a biomarker for oral cancers in early stages (I and II). The systematic literature search identified studies reporting the diagnostic accuracy of salivary MMP-9 levels. The review of included studies suggested a promising potential of salivary MMP-9 for the early detection of oral cancers, specifically at initial stages I and II.

The included studies in the present review were from Europe, Asia, and Africa. Qualitative assessment is the most critical component of any systematic review, facilitating the synthesis of high-quality evidence [38]. In line with this, studies included for assessment are expected to provide all the necessary information to establish constructive results. The DTA studies should be reported according to Standards for Reporting Diagnostic Accuracy Studies (STARD) guidelines [39] to ensure every aspect gets covered. However, all studies in this review were surprisingly not per STARD guidelines.

The QUADAS-2 tool was used for qualitative assessment. It presented a high risk of bias, however, with low concerns for applicability. This is because all the studies included in this review were exploratory design according to Cochrane Diagnostic Test Accuracy (DTA) guidelines [29]. This guideline categorizes diagnostic accuracy studies as exploratory and pragmatic studies. The exploratory studies establish a hypothesis, whereas pragmatic trials help in clinical decision-making. Another difference between these studies is that the threshold value is explored in the exploratory study, whereas it is pre-specified in the pragmatic study. The exploratory study often uses a smaller sample size and involves a single centre compared to a larger sample size and multiple centers in pragmatics [28]. The pragmatic designs have a single criterion for patient selection, which is also called a single gate study. Second, a blinded interpretation of the index and reference test is sought [40–43]. Third, the flow and timing of both tests are essential because any long interval between two or more tests can introduce bias in a study [40–43]. Ideally, both tests shall be taken simultaneously [40–43].

The sensitivity and specificity forest plots (Fig. 5) summarize the results of five studies on the oral cancer diagnosis using MMP-9 as a biomarker. All five studies 24, 26) demonstrate a sensitivity of 1.00, indicating that the test correctly identified all actual positive cases in each study. The 95% confidence intervals (CIs) for sensitivity in all studies range from 0.78 to 1.00, suggesting high sensitivity with some variability in the lower bound of the CI. The consistency of these results across multiple studies suggests that the diagnostic test using MMP-9 as a biomarker is highly effective for detecting oral cancer in its early stages (I and II). The subgroup analyses were not possible as MMP-9 values were not consistently reported by included studies. One study report values by cancer staging [24] and others [22, 26] by grading, only one study reports according to anatomical subsite [24] (Table S1).

However, the overall effect shown in Fig. 4 challenges the hypothesis that MMP-9 could serve as a diagnostic biomarker. There can be several reasons why the overall effect’s p-value is not significant, even though individual studies show substantial p-values. First, high heterogeneity represents high variability among studies, diluting the overall impact and making it less likely to reach statistical significance. Secondly, although the overall sample size was large, the effect sizes varied significantly between studies. The inconsistent effect sizes across studies can result in the overall effect not being statistically significant. Thirdly, wide confidence intervals in overall effect size indicate uncertainty in the estimate, which can lead to insignificant p-values. Other reasons may include the presence of publication bias and the use of different statistical methods across studies. Additionally, while our meta-analysis aimed to provide pooled estimates, the presence of this heterogeneity suggests that caution must be exercised in interpreting summary estimates and underscores the need for further research to verify the diagnostic performance of MMP-9 in OSCC.

Beyond numerical accuracy, the clinical applicability of an index test is evaluated in terms of false positives and false negatives. In diagnostic settings where misclassification can have serious consequences, understanding the trade-offs between sensitivity and specificity is crucial. False negatives may lead to delayed interventions and potentially worsen clinical outcomes, while false positives could result in unnecessary treatments and increased healthcare costs. Therefore, the clinical utility of the test must be evaluated beyond statistical measures, integrating real-world implications.

The study has several limitations. Although, the literature search was systematic, missing of any published or unpublished article cannot be ruled out. Secondly, the included studies were found to have low methodological quality and level of reporting. While these two-tailed designs might be helpful in early evaluation of diagnostic tests, they usually lead to inflated test results compared to what could be expected in clinical practice. Lastly, since there was high level of heterogeneity i.e. I^2^ = 100% among the studies, the summary estimates from the exploratory meta-analysis need to be interpreted very cautiously. Further studies on pragmatic design are needed to establish its importance as an aid in screening oral cancers.

Future research should aim to refine threshold selection strategies, ensuring better test standardization and reproducibility across diverse clinical settings. Additionally, salivary MMP-9 testing can be seamlessly integrated into clinical workflow through the development of rapid, non-invasive point of care assays and chairside diagnostic kits. Apart from its use as POC diagnostic tool, salivary MMP-9 can be used as a screening tool and incorporated into high-risk population programs such as tobacco users or individuals with precancerous lesions using mobile health care units or saliva collection kits. Such integration would support timely intervention, personalized care and improved outcomes. Besides, comparison with other established biomarkers is essential for placing its performance in proper clinical context. By directly assessing multiple biomarkers under identical conditions and patient cohorts, clinicians can identify synergistic biomarker panels which when used together to enhance diagnostic accuracy beyond what any single marker can achieve.

## Conclusions

Within the limitations of this review, salivary MMP-9 levels demonstrate promising potential as a critical biomarker for the detection of OSCC in early stages (I, II). Elevated MMP-9 in saliva may reflect early pathological changes preceding overt clinical symptoms. Its testing at the time of initial inquiry of OPMD will offer a valuable window for timely intervention. To fully validate and harness this potential, future research should prioritize prospective multicentre studies to ensure reproducibility across diverse populations and clinical settings.

## Supplementary Information

Supplementary Material 1.

Supplementary Material 2.

## Data Availability

The data of this study is available from the corresponding author upon reasonable request.

## Abbreviations

AUC: Area Under the Curve
CT-PET: Computed Tomography- Positron Emission Tomography
DTA: Diagnostic Test Accuracy
ECM: Extra Cellular Matrix
ELISA: Enzyme Linked Immunosorbent Assay
LOC: Lip and Oral cavity Cancer
MMP: Matrix Metallo Proteinases
OPMD: Oral Pre-Malignant Disease
OSCC: Oral Squamous Cell Carcinoma
QUADAS: Quality Assessment Tool for Diagnostic Accuracy Studies
REVMAN: Review Manager
ROC: Receivers Operators Curve
SEN: Sensitivity
SPE: Specificity
STARD: Standards for Reporting Diagnostic Accuracy Studies
USWS: Un Stimulated Whole Saliva

## References

[1] Bray F, Laversanne M, Sung H, Ferlay J, Siegel RL, Soerjomataram I, et al. Global cancer statistics 2022: GLOBOCAN estimates of incidence and mortality worldwide for 36 cancers in 185 countries. CA Cancer J Clin. 2024;74(3):229–63. 38572751 10.3322/caac.21834

[2] Miranda-Filho A, Bray F. Global patterns and trends in cancers of the lip, tongue and mouth. Oral Oncol. 2020;102:104551. 31986342 10.1016/j.oraloncology.2019.104551

[3] Huang J, Chan SC, Ko S, Lok V, Zhang L, Lin X, et al. Disease burden, risk factors, and trends of lip, oral cavity, pharyngeal cancers: a global analysis. Cancer Med. 2023;12(17):18153–64. 37519070 10.1002/cam4.6391 PMC10524054

[4] Piyarathne NS, Rasnayake RMSGK, Angammana R, Chandrasekera P, Ramachandra S, Weerasekera M, et al. Diagnostic salivary biomarkers in oral cancer and oral potentially malignant disorders and their relationships to risk factors – a systematic review. Expert Rev Mol Diagn. 2021;21(8):789–807. 34148471 10.1080/14737159.2021.1944106

[5] Ulaganathan G, Mohamed Niazi KT, Srinivasan S, Balaji VR, Manikandan D, Hameed KAS, et al. A clinicopathological study of various oral cancer diagnostic techniques. J Pharm Bioallied Sci. 2017;9(Suppl 1):S4–10. 29284926 10.4103/jpbs.JPBS_110_17 PMC5731041

[6] Chasma F, Pedr King R, Ker SY. Are there diagnostic alternatives to histopathology in detecting oral cancer? Evid Based Dent. 2022;23(1):24–5. 35338323 10.1038/s41432-022-0251-1

[7] Abati S, Bramati C, Bondi S, Lissoni A, Trimarchi M. Oral cancer and precancer: a narrative review on the relevance of early diagnosis. Int J Environ Res Public Health. 2020;17(24):9160. 33302498 10.3390/ijerph17249160 PMC7764090

[8] Zabin Alotaibi K, Hameed Kolarkodi S. Effectiveness of adjunctive screening tools for potentially malignant oral disorders and oral cancer: a systematic review. Saudi Dent J. 2024;36(1):28–37. 38375389 10.1016/j.sdentj.2023.10.011 PMC10874794

[9] Strimbu K, Tavel JA. What are biomarkers? Curr Opin HIV AIDS. 2010;5(6):463–6. 20978388 10.1097/COH.0b013e32833ed177 PMC3078627

[10] Silva LTL, Nicolau CA, Moreira CSPD, Silva AA, de Carvalho L, Fernandes GE. RMD, Diagnosis of oral cancer using biomarkers, a non invasive proposal | International Seven Journal of Health Research. Available from: https://sevenpublicacoes.com.br/index.php/ISJHR/article/view/3748. Cited 19 Mar 2025.

[11] Sahibzada HA, Khurshid Z, Khan RS, Naseem M, Siddique KM, Mali M, et al. Salivary IL-8, IL-6 and TNF-α as potential diagnostic biomarkers for oral cancer. Diagnostics. 2017 June;7(2):21. 10.3390/diagnostics7020021 PMC5489941 28397778

[12] Melguizo-Rodríguez L, Costela-Ruiz VJ, Manzano-Moreno FJ, Ruiz C, Illescas-Montes R. Salivary Biomarkers and Their Application in the Diagnosis and Monitoring of the Most Common Oral Pathologies. Int J Mol Sci. 2020;21(14):5173. 32708341 10.3390/ijms21145173 PMC7403990

[13] Khurshid Z, Zafar MS, Khan RS, Najeeb S, Slowey PD, Rehman IU. Role of Salivary Biomarkers in Oral Cancer Detection. In: Advances in Clinical Chemistry. Elsevier; 2018. pp. 23–70. Available from: https://linkinghub.elsevier.com/retrieve/pii/S0065242318300313. Cited 19 Apr 2025. 10.1016/bs.acc.2018.05.002 30144841

[14] Das S, Dey MK, Devireddy R, Gartia MR. Biomarkers in cancer detection, diagnosis, and prognosis. Sensors. 2024;24(1):37. 10.3390/s24010037 PMC10780704 38202898

[15] Mondal S, Adhikari N, Banerjee S, Amin SA, Jha T. Matrix metalloproteinase-9 (MMP-9) and its inhibitors in cancer: a minireview. Eur J Med Chem. 2020;194:112260. 32224379 10.1016/j.ejmech.2020.112260

[16] Monea M, Pop AM. The use of salivary levels of matrix metalloproteinases as an adjuvant method in the early diagnosis of oral squamous cell carcinoma: a narrative literature review. Curr Issues Mol Biol. 2022;44(12):6306–22. 36547091 10.3390/cimb44120430 PMC9776994

[17] Cabral-Pacheco GA, Garza-Veloz I, la Castruita-De Rosa C, Ramirez-Acuña JM, Perez-Romero BA, Guerrero-Rodriguez JF, et al. The roles of matrix metalloproteinases and their inhibitors in human diseases. Int J Mol Sci. 2020;21(24):9739. 33419373 10.3390/ijms21249739 PMC7767220

[18] AlAli AM, Walsh T, Maranzano M. CYFRA 21 – 1 and MMP-9 as salivary biomarkers for the detection of oral squamous cell carcinoma: a systematic review of diagnostic test accuracy. Int J Oral Maxillofac Surg. 2020;49(8):973–83. 32035907 10.1016/j.ijom.2020.01.020

[19] Utilization of the PICO framework. to improve searching PubMed for clinical questions | BMC Medical Informatics and Decision Making | Full Text. Available from: https://bmcmedinformdecismak.biomedcentral.com/articles/. 10.1186/1472-6947-7-16. Cited 26 Apr 2025 Apr 26. 10.1186/1472-6947-7-16 PMC1904193 17573961

[20] Plana MN, Arevalo-Rodriguez I, Fernández-García S, Soto J, Fabregate M, Pérez T, et al. Meta-DiSc 2.0: a web application for meta-analysis of diagnostic test accuracy data. BMC Med Res Methodol. 2022;22(1):306. 36443653 10.1186/s12874-022-01788-2 PMC9707040

[21] Ghallab NA, Shaker OG. Serum and salivary levels of chemerin and MMP-9 in oral squamous cell carcinoma and oral premalignant lesions. Clin Oral Invest. 2017;21(3):937–47. 10.1007/s00784-016-1846-8 27161218

[22] Smriti K, Ray M, Chatterjee T, Shenoy RP, Gadicherla S, Pentapati KC, et al. Salivary MMP-9 as a biomarker for the diagnosis of oral potentially malignant disorders and oral squamous cell carcinoma. Asian Pac J Cancer Prev. 2020;21(1):233. 31983189 10.31557/APJCP.2020.21.1.233 PMC7294014

[23] Peisker A, Raschke GF, Fahmy MD, Guentsch A, Roshanghias K, Hennings J, et al. Salivary MMP-9 in the detection of oral squamous cell carcinoma. Med Oral Patol Oral Cir Bucal. 2017;22(3):e270. 28160595 10.4317/medoral.21626 PMC5432074

[24] Shin YJ, Vu H, Lee JH, Kim HD. Diagnostic and prognostic ability of salivary MMP-9 for oral squamous cell carcinoma: a pre-/post-surgery case and matched control study. PLoS One. 2021;16(3):e0248167. 33735248 10.1371/journal.pone.0248167 PMC7971541

[25] Thiruvalluvan A, Reddy JRC, Sekizhar V, Subramanyam V. Estimation of salivary matrix metalloproteinase-9 in oral leukoplakia, oral submucous fibrosis, and healthy individuals: a comparative observational study. J Stoma. 2021;74(4):221–6.

[26] Pazhani J, Chanthu K, Jayaraman S, Varun BR. Evaluation of salivary MMP-9 in oral squamous cell carcinoma and oral leukoplakia using ELISA. J Oral Maxillofac Pathol. 2023;27(4):649. 38304520 10.4103/jomfp.jomfp_426_23 PMC10829443

[27] Kochurova EV, Nikolenko VN. Estimation of expression of oral fluid biomarkers in the diagnosis of pretumor diseases of oral mucosa. Bull Exp Biol Med. 2017;163(1):87–91. 28580490 10.1007/s10517-017-3744-8

[28] Bossuyt PM. Understanding the design of test accuracy studies. In: Deeks JJ, Bossuyt PM, Leeflang MM, Takwoingi Y, editors. Cochrane Handbook for Systematic Reviews of Diagnostic Test Accuracy. 1st edn Wiley; 2023. pp. 35–51. Available from: https://onlinelibrary.wiley.com/doi/. 10.1002/9781119756194.ch3. Cited 21 Feb 2025.

[29] Bossuyt PM, Olsen M, Hyde C, Cohen JF. An analysis reveals differences between pragmatic and explanatory diagnostic accuracy studies. J Clin Epidemiol. 2020;117:29–35. 31561014 10.1016/j.jclinepi.2019.09.017

[30] Whiting P, Rutjes AW, Reitsma JB, Bossuyt PM, Kleijnen J. The development of QUADAS: a tool for the quality assessment of studies of diagnostic accuracy included in systematic reviews. BMC Med Res Methodol. 2003;3(1):25. 14606960 10.1186/1471-2288-3-25 PMC305345

[31] Bobbitt Z. Cohen’s Kappa Calculator. Statology. 2021. Available from: https://www.statology.org/cohens-kappa-calculator/. Cited 22 Sept 2024.

[32] Huang H. Matrix metalloproteinase-9 (MMP-9) as a cancer biomarker and MMP-9 biosensors: recent advances. Sensors. 2018;18(10):3249. 30262739 10.3390/s18103249 PMC6211011

[33] Yabluchanskiy A, Ma Y, Iyer RP, Hall ME, Lindsey ML. Matrix metalloproteinase-9: many shades of function in cardiovascular disease. Physiology (Bethesda). 2013;28(6):391–403. 24186934 10.1152/physiol.00029.2013 PMC3858212

[34] Shaw AK, Garcha V, Shetty V, Vinay V, Bhor K, Ambildhok K, et al. Diagnostic accuracy of salivary biomarkers in detecting early oral squamous cell carcinoma: a systematic review and meta-analysis. Asian Pac J Cancer Prev. 2022;23(5):1483–95. 35633529 10.31557/APJCP.2022.23.5.1483 PMC9587865

[35] Khijmatgar S, Yong J, Rübsamen N, Lorusso F, Rai P, Cenzato N, et al. Salivary biomarkers for early detection of oral squamous cell carcinoma (OSCC) and head/neck squamous cell carcinoma (HNSCC): a systematic review and network meta-analysis. Jpn Dent Sci Rev. 2024;60:32–9. 38204964 10.1016/j.jdsr.2023.10.003 PMC10776379

[36] Aro K, Kaczor-Urbanowicz K, Carreras-Presas CM. Salivaomics in oral cancer. Curr Opin Otolaryngol Head Neck Surg. 2019;27(2):91–7. 30507690 10.1097/MOO.0000000000000502

[37] Papale F, Santonocito S, Polizzi A, Giudice AL, Capodiferro S, Favia G, et al. The new era of salivaomics in dentistry: frontiers and facts in the early diagnosis and prevention of oral diseases and cancer. Metabolites. 2022;12(7):638. 35888762 10.3390/metabo12070638 PMC9319392

[38] Ochodo EA, van Enst WA, Naaktgeboren CA, de Groot JA, Hooft L, Moons KG, et al. Incorporating quality assessments of primary studies in the conclusions of diagnostic accuracy reviews: a cross-sectional study. BMC Med Res Methodol. 2014;14(1):33. 24588874 10.1186/1471-2288-14-33 PMC3942773

[39] Cohen JF, Korevaar DA, Altman DG, Bruns DE, Gatsonis CA, Hooft L, et al. STARD 2015 guidelines for reporting diagnostic accuracy studies: explanation and elaboration. BMJ Open. 2016;6(11):e012799. 28137831 10.1136/bmjopen-2016-012799 PMC5128957

[40] Buehler A, Ascef B, Junior H, Ferri C, Fernandes J. Rational use of diagnostic tests for clinical decision making. Rev Assoc Med Bras. 2019;65:452–9. 30994847 10.1590/1806-9282.65.3.452

[41] Chassé M, Fergusson DA. Diagnostic accuracy studies. Semin Nucl Med. 2019;49(2):87–93. 30819399 10.1053/j.semnuclmed.2018.11.005

[42] Deeks JJ, Bossuyt PM. Evaluating medical tests. In: Deeks JJ, Bossuyt PM, Leeflang MM, Takwoingi Y, editors. Cochrane Handbook for Systematic Reviews of Diagnostic Test Accuracy. 1st edn Wiley; 2023. pp. 19–33. Available from: https://onlinelibrary.wiley.com/doi/. 10.1002/9781119756194.ch2. Cited 21 Feb 2025.

[43] Deeks JJ, Takwoingi Y, Macaskill P, Bossuyt PM. Understanding test accuracy measures. In: Deeks JJ, Bossuyt PM, Leeflang MM, Takwoingi Y, editors. Cochrane Handbook for Systematic Reviews of Diagnostic Test Accuracy. 1st edn Wiley; 2023. pp. 53–72. Available from: https://onlinelibrary.wiley.com/doi/. 10.1002/9781119756194.ch4. Cited 24 Jul 2024.

