# Supplementary material for: MMP-9 as a diagnostic salivary biomarker for early detection of oral cancers: systematic review and meta-analysis
Source: BMC Oral Health. 2026 Jan 3;26:219. doi: 10.1186/s12903-025-07513-x (PMC12866002; doi:10.1186/s12903-025-07513-x)
Supplement: Supplementary file 2 — Supplementary Material 2. [file 12903_2025_7513_MOESM2_ESM.docx]

Table S 1: Table summarizing stage and subsite characteristics of included studies

|  |  | Stage /grade | | Subsite | MMP-9 |
| --- | --- | --- | --- | --- | --- |
| Ghallab and Shaker  2017 | **OSCC** | Early | | - Tongue - Retromolar - Labial mucosa - Buccal mucosa - Floor of mouth | NR |
|  | **OPML** | - Atrophic lichen planus, - Actinic keratosis - Speckled leukoplakia   (Moderate to severe) | |  | NR |
| Kochurova  et al  2017 | **OPML** | Papilloma 66.7  Leucoplakia 21.4  Cutaneous horn 11.9 | | Oral mucosa (66.7%),  tongue (33.3%),  Lips (22.2%)  Jaw (11.1%) | NR |
| Pazhani et al  2023 | **OSCC** | **Well**  **Differentiated**  58.8%,  **Moderately differentiated**  26.5%  **Poorly differentiated**  14.7% | | Buccal mucosa 52%,  Tongue 23%  Vestibule 8%,  Gingiva 5%,  Alveolar ridge 6%  Lips 3%.  Floor of the mouth 3%. | **Well**  **Differentiated**  49.0 ± 2.2 ng/ml.  **Moderately differentiated**  48.7 ± 0.9 ng/ml.  **Poorly differentiated**  62.7 ± 6.4 ng/ml. |
|  | **OPML** | **Oral leukoplakia**  Mild dysplasia  26.5%  Moderate dysplasia 41.2%,  Severe dysplasia 32.4%. | | Buccal mucosa 62%,  Tongue 22%,  Labial mucosa13%  Gingiva 3% | Mild dysplasia  4.7 ng/ml  Moderate dysplasia  3.3 ng/ml  severe dysplasia  4.9 ng/ml. |
| Peiskar et al  2017 | **OSCC** | G1- 1  G2- 20  G3- 8  G4 -1 | **T stage**  T1- 10  T2- 6  T3- 3  T4- 1  **N Stage**  N0- 15  N1- 6  N2- 9  **M Stage**  M0- 27  M1- 3 | Oral floor 6  Tongue 4  Upper jaw 5  Lower jaw 2  Buccal mucosa 2  Soft palate 2  Multiple areas 9 | NR |
| Shin et al  2021 | **OSCC** | Stage 0 (2.8%)  Stage 1 (18.9%)  Stage 2 (33.0%)  Stage 3 (10.4%)  Stage 4 (34.9%) | | **Floor of mouth** (3.8%)  **Tongue**  (21.7%)  **Buccal mucosa** (17.0%)  **Alveolar ridge** (45.3%)  **Hard palate** (4.7%)  **Retromolar** (7.5%) | Stage 0- 395.11  Stage 1- 332.49  Stage 2- 842.68  Stage 3- 1029.8  Stage 4- 524.38 |
|  |  |  |  |  | **Floor of mouth**  882.74  **Tongue**  531.66  **Buccal mucosa** 759.29  **Alveolar ridge** 468.96  **Hard palate** 1103.57  **Retromolar**  505.34 |
| Smriti et al  2020 | **OSCC** | **Well-differentiated**  **Moderately differentiated**    **Poorly differentiated** | | Buccal  mucosa 25  Retromolar area 4  Tongue 4  Hard palate 2  Floor of mouth 2  Lower lip 3  Alveolus 5 | **Well-differentiated**  353.65±108.47  **Moderately differentiated**  342.82±74.27  **Poorly differentiated** 506.78±134.37 |
|  | **OPML** | Erythroplakia 8  Leukoplakia 5  OSMF 7 | |  | 406.28±154.23  299.09±99.69  293.08±39.58 |
| Thiruvalluvan et al  2021 | **OPML** | OSMF 20  leukoplakia 20 | | NR | 9.42 ± 2.882  10.59 ± 2.862 |
